# *Ex Vivo* Behaviour of Human Bone Tumor Endothelial Cells

**DOI:** 10.3390/cancers5020404

**Published:** 2013-04-11

**Authors:** Teresa Infante, Elena Cesario, Michele Gallo, Flavio Fazioli, Annarosaria De Chiara, Cristina Tutucci, Gaetano Apice, Filomena de Nigris

**Affiliations:** 1 SDN-Foundation, Institute of Diagnostic and Nuclear Development, IRCCS, 80143 Naples, Italy; 2 Department of Biochemistry and Biophysics, Second University of Naples, 80138 Naples, Italy; 3 Division of Skeletal Muscles Oncology Surgery, National Cancer Institute, Pascale Foundation, 80131 Naples, Italy; 4 Anatomic Pathology Unit, National Cancer Institute, Pascale Foundation, 80131 Naples, Italy; 5 Medical Oncology of Bone and Soft Sarcoma tissues Unit, National Cancer Institute, Pascale Foundation, 80131 Naples, Italy

**Keywords:** sarcomas, angiogenesis, endothelial cells, tumor microenvironment

## Abstract

Cooperation between endothelial cells and bone in bone remodelling is well established. In contrast, bone microvasculature supporting the growth of primary tumors and metastasis is poorly understood. Several antiangiogenic agents have recently been undergoing trials, although an extensive body of clinical data and experimental research have proved that angiogenic pathways differ in each tumor type and stage. Here, for the first time, we characterize at the molecular and functional level tumor endothelial cells from human bone sarcomas at different stages of disease and with different histotypes. We selected a CD31^+^ subpopulation from biopsies that displayed the capability to grow as adherent cell lines without vascular endothelial growth factor (VEGF). Our findings show the existence in human primary bone sarcomas of highly proliferative endothelial cells expressing CD31, CD44, CD105, CD146 and CD90 markers. These cells are committed to develop capillary-like structures and colony formation units, and to produce nitric oxide. We believe that a better understanding of tumor vasculature could be a valid tool for the design of an efficacious antiangiogenic therapy as adjuvant treatment of sarcomas.

## 1. Introduction

The unique characteristics of bone provide homing signals to different types of cancer cells, which together with biochemical (e.g., cytokines, growth factors) and physical (e.g., acidic pH, high extracellular calcium concentration) properties of the bone create an advantageous microenvironment for primary tumors and metastasis growth [1,2]. Efforts directed toward targeting tumor microenvironment for solid tumor therapy have largely focused on blocking blood vessels [3,4]. However, to date, the promising results reported in preclinical studies have not been reproduced in the clinical setting [5]. The lack of markers for monitoring antiangiogenic therapy and the fact that tumor endothelial cells (TECs) are different from normal endothelial cells (ECs) have contributed to short-lived response to treatment [6]. Identifying markers and overcoming the mechanisms that mediate resistance to antiangiogenic drugs will likely yield improved clinical outcomes. Several studies have shown that TECs differ from normal ECs in the expression of specific surface markers and genetic abnormalities responsible for incomplete, irregular and tortuous blood vessels with increased permeability [1,2,7,8]. These properties derive from tumor type, blood vessels site, and stage of tumor progression governed by extracellular environment including biochemical and signalling pathways as well as by cellular context [9]. 

The phenotypic and genetic heterogeneity of TECs raises the issue of whether they originate from normal ECs, bone marrow, resident stem cells, transdifferentiation or de-differentiation of tumor stem cells into ECs, or cell fusion between cancer cells and normal ECs [1,10]. Furthermore, the mechanism by which TECs acquire a constitutive proangiogenic phenotype and resistance to common antiangiogenic drugs is still unknown [11,12,13]. Bone sarcomas are a group of mesenchymal malignancies, highly vascularized with many different subtypes each with unique clinical pathological features [14]. To improve the outcome of unresectable, chemoresistant or metastatic patients, ongoing trials are testing antiangiogenic drugs as adjuvant therapy [15]. However, not all subtypes are expected to respond since bone tumor microenvironments are poorly understood and no data are available on the phenotypic and functional characteristics of TECs. Understanding the origin of bone TECs as well as their molecular and functional characteristics may help to develop appropriate and specific antiangiogenic therapies. Here, we characterize for the first time human bone TECs from fresh biopsies at functional and molecular levels.

## 2. Experimental Section

### 2.1. Human Tissue Samples

Tissues, surgically resected from 13 cases, were clinically classified as bone and soft sarcoma by histologic and immunohistologic criteria, according to UICC/AJCC by Istituto Nazionale Tumori, Fondazione “G. Pascale” (Naples, Italy), under Local Ethical Committee approval. Specimens were finely minced with scissors and then digested by incubation for 1 h at 37 °C in Dulbecco’s modified Eagle’s medium (DMEM) containing collagenase II (Sigma Chemical, Perth, WA, USA). After washings in medium plus 10% FCS (GIBCO, Grand Island, NY, USA), cell suspension was forced through a graded series of meshes to separate the cell components from stromal cells and aggregates. Cells were pelleted, resuspended, and isolated using anti-CD31 Ab coupled to magnetic beads, with the MACS system (Miltenyi Biotec, Auburn, CA, USA). Briefly, 2 × 10^5^ cells/μL were labelled with the anti-CD31 mAb for 20 min and then were washed twice and resuspended in MACS buffer (PBS without Ca^2+^ and Mg^2+^, supplemented with 1% bovine serum albumin (BSA) and 5 mmol/L EDTA). After washings, cells were separated on a magnetic stainless steel wool column (Miltenyi Biotec.) according to the manufacturer’s recommendations. CD31^+^ cells were plated onto 0.1% gelatin and were grown in EGM-2 medium (GIBCO) supplemented with epidermal growth factor (10 ng/mL), hydrocortisone (1 µg/mL), and 0.5% FCS (GIBCO). Lack of contaminating leukocytes or of smooth muscle cells was verified by FACS analysis with anti-CD45 mAb and anti-smooth muscle actin Ab. Human aortic endothelial cells (HAEC) were obtained from ATCC, as described previously [16,17].

### 2.2. Cytofluorimetric Analysis

Cells were detached from plates with non-enzymatic cell dissociation solution, washed in PBS containing 2% BSA to block remaining non-specific sites. 2 × 10^5^ cells were then incubated for 30 min at 4 °C with the appropriate Ab or with the relevant control in PBS containing 2% BSA. For cytofluorimetric analysis the primary antibodies used were: anti CD31-FITC (R&D Systems, Inc. Minneapolis, MN, USA), anti VE-cadherin-PE (Santa Cruz Biotechnology, Inc. Milan, Italy), anti CD133-PE (Miltenyi Biotech), anti fusin-PE (Santa Cruz Biotechnology), anti CD14-PE (R&D Systems), anti CD44-FITC (R&D Systems), anti CD13-PE/Cy5 (Chemicon International, Temecula, CA, USA), anti CD34-PE, anti CD105-PE, anti CD45-PE, anti CD90-PE, anti CD131-PE, anti CCR7-PE, anti CD133-PE (Miltenyi Biotech.), anti CD146-PE (Miltenyi Biotech.) and anti CD309-PE (Miltenyi Biotech.). Cells were analyzed on a FACS Vantage cell sorter (Becton Dickinson, Franklin Lakes, NJ, USA) and 30,000 event cells were analyzed at each experimental point. Control experiments included incubation with isotopic human IgG (Becton Dickinson). Data were analyzed using CellQuest software (Becton Dickinson).

### 2.3. Immunofluorescence Studies

For immunofluorescence 2 × 10^4^ cells were grown on cover slides, fixed in 1% paraformaldehyde and first permeabilized with 0.1% Triton and then incubated with mouse anti-human CD31 (Chemicon International) or goat anti-human von-Willebrand (AbCam) at 4 °C for 45 min. Cells were then stained by addition of FITC conjugated mouse anti-goat IgG or goat anti-mouse labelled with Texas red or Alexa Fluor 488 (Molecular Probe, Invitrogen, Milan, Italy), at 1:1,000 dilution. Cell nuclei were stained with DAPI (Sigma). Confocal microscopy analysis was performed using a Zeiss confocal microscope, (Carl Zeiss International, Berlin, Germany).

### 2.4. Nitric Oxide and Platelet Activating Factor (PAF) Assays

Nitric oxide (NO) production was detected using QuantiChrom TM Nitric Oxide Assay Kit (D2NO-100; Bioassay, San Diego, CA, USA). Cells were incubated for 60 min at 37 °C in the working solution and then absorbance was read at 540 nm. Cells were stimulated with acetylcholine in the absence or presence of the NO synthase inhibitor nitro-L-arginine methyl ester (L-NAME) (1 mM), and, as a control, with L-arginine (1 mM) (Bioassay). Three different experiments were performed at 5th and 13th passages. Platelet Activating Factor (PAF) assay was performed using Platelet Activating Factor (PAF) ELISA Kit (Cayman Chemical, Ann Arbor, MI, USA) following the manufacturer’s instructions.

### 2.5. Cell Proliferation Assay

2 × 10^4^ cells/well were seeded into 24-well plates in EGM-2 containing FCS ranging between 0.5–10% or plus growth factors (GIBCO). After 24, 48 and 72 h of incubation under the appropriate conditions, monolayers were carefully washed, dried, and treated with 0.75% crystal violet in a solution of 50% ethanol, 0.25% NaCl, and 1.75% formaldehyde. After washing, the dye was eluted with 1% SDS in PBS, and absorbance was read at 595 nm with an ELISA reader (TECAN SPECTRA Fluor Plus fluorescence, absorbance, and luminescence reader, MTX Lab Systems, Inc., Vienna, VI, USA). Cell numbers were determined on the basis of a standard curve obtained with known cell numbers. All experiments were performed in triplicate. In addition, DNA synthesis was detected as incorporation of 5-bromo-2'-deoxyuridine (BrdU) into cellular DNA by using an ELISA kit (Roche Molecular Biochemicals, Basel, Switzerland), according to the manufacturer’s instructions. Briefly, 10 μM BrdU was added to the stimulated cells for 18 h. Cells were then fixed with 0.5 M ethanol-HCl and incubated with nuclease to digest the DNA. BrdU incorporated into the DNA was detected by using an anti-BrdU peroxidase-conjugated mAb and was visualized with a soluble chromogenic substrate. Optical density was measured with the ELISA reader at 450 nm.

### 2.6. Tube Formation and Migration Assay

*In vitro* angiogenesis assay was performed on growth factor-reduced Matrigel diluted 1:1 with cold EGM-2 minimum media. HAECs or TECs (1 × 10^4^ cells/well) were seeded onto Matrigel-coated wells (12 multiwell plate) cultured with EGM-2 medium without growth factors containing 0.25% BSA. Additionally, migration assay was performed growing 1 × 10^4^ cells in EGM-2 medium without growth factors in presence of 10 ng/mL SDF-1 (R&D Systems). The capillary-like tubule structures in each well were photographed with Nikon light microscope, and experimental results were recorded. The mean of tube length/μm × field (3 different fields) was calculated with the MicroImage analysis system (Cast Imaging srl, Venice, Italy).

### 2.7. Statistical Analysis

Student t-test (two-tailed) was used for statistical evaluation. Level of significance was set at *p* < 0.05.

## 3. Results

### 3.1. Characterization of TECs

We examined 13 human bone sarcoma biopsies (with different clinical and histologic characteristics as indicated in Table 1) obtained after surgical resection at the Istituto Nazionale Tumori “G. Pascale”, Naples (Italy). 

**Table 1:** Clinical pathological features of bone biopsies.

Case	Gender	Age	Histotype	Grade
1	M	30	GCT *	I
2	M	25	Chondrosarcoma	II
3	M	40	Fibrosarcoma	I
4	F	35	Osteosarcoma	II
5	F	45	Chondrosarcoma	II
6	F	23	Chondrosarcoma	II
7	M	43	Osteosarcoma	III
8	F	31	Fibrosarcoma	II
9	M	26	Osteosarcoma	II
10	M	27	GCT *	I
11	F	22	Chondrosarcoma	II
12	F	20	GCT *	I
13	F	45	GCT *	I

* GCT: Giant cell tumor of bone.

**Figure 1:**
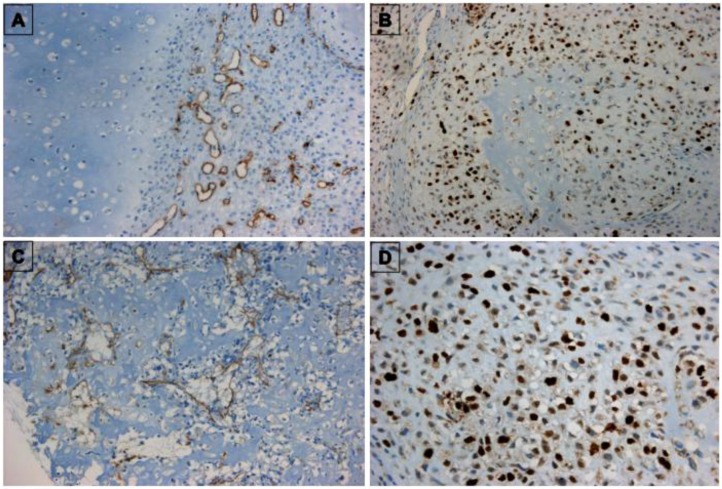
Immunohistochemistry of bone sarcomas. (**A**) CD31-immunostained chondrosarcoma specimen (TEC 1 line); (**B**) ki67-stained chondrosarcoma specimen; (**C**) CD31-stained osteosarcoma specimen (TEC 2 line); (**D**) Ki67-stained osteosarcoma specimen.

**Figure 2:**
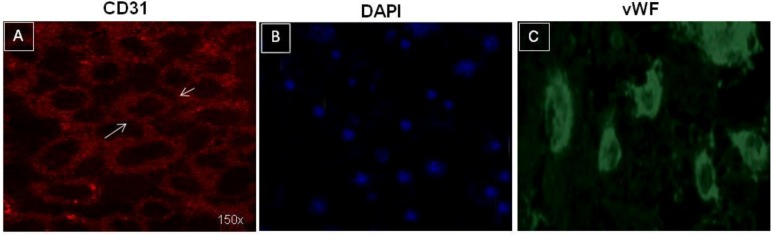
Cultured tumor endothelial cells from bone sarcoma. (**A**) Confluent tumor endothelial cells (TEC 1 line) stained with CD31, exhibiting the typical cobblestone structure (Panel A); (**B**) DAPI control staining; (**C**) Tumor endothelial cells TEC 1 stained with von Willebrand factor (cytoplasmatic staining).

A subpopulation of CD31^+^ cells (a marker of endothelial cells) was selected from all biopsies by using magnetic beads coupled to CD31 antibody. We chose CD31 antibody because immunohistochemistry data on the same biopsies indicated that CD31 was expressed by vascular structures but not by tumor cells (Figure 1). After selection, TECs were established and grown without VEGF as adherent cells and were able to continue for several culture passages. Although at the first passage all cells lines were positive to CD31 antibody (Figure 2A,B), long-term culturing determined a reduction of CD31 (see below) and vWF positivity (Figure 2C). 

**Figure 3:**
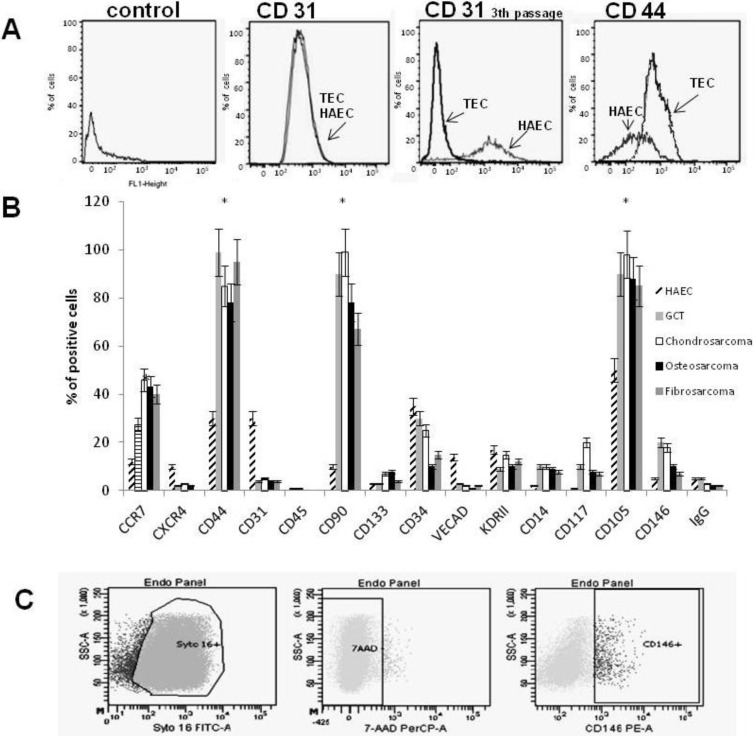
Characterization of TECs. (**A**) Expression of endothelial markers in a representative TEC line and HAEC cells, used as a control detected by FACS analysis. FACS analysis of HAECs, and TEC 1, with CD31 antibody at 1st passage and 3th passage. Expression of CD44 antigen in HAECs and TEC 1. (**B**) Bar graph expressing percentage of positive cells to specific antigens analyzed by FACS. Data are expressed as mean ± SE of 10 different TEC lines grouped by histology and three control cell lines. Each experiment was repeated three times. TECs *vs*. HAECs (*p* < 0.001). IgG was used as negative control. (**C**) Representative example of TECs analyzed with CD146 antibody.

In addition, TECs showed a normal karyotype indicating they were not contaminated or originating from tumor cells (data not shown). TECs from different biopsies were subjected to cytofluorimetric analysis with endothelial markers and staminal markers, showing heterogeneous positivity. However, all TEC cell lines were strongly positive to CD105, CD90 and CD146 compared to normal ECs (Figure 3B) [18]. During culturing a lower expression of endothelial markers such as CD31, CD309 (VEGF receptor) and VE-Cadherin as well as mesenchymal markers such as CD117 and CD73 (ranging between 1–2% of positive cells) was detected in TECs compared to the same cells at first passage (Figure 3B) consistent with cell growth in plate [19]. In contrast, 98% of TECs expressed CD44 (compared to 30% of HAECs), a marker of invasiveness possibly involved in cell motility. Concomitantly, TECs were negative to CD45, CD14 and smooth muscle actin Ab, suggesting non-contamination from bone-marrow cells, leukocytes and smooth muscle cells [20].

### 3.2. *In Vitro* Behaviour of TECs

**Figure 4:**
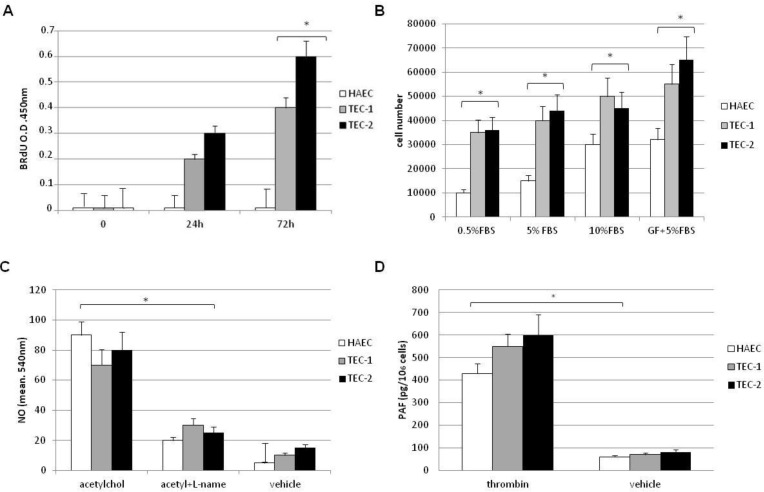
Growth and functional characterization of TECs. (**A**) BrdU incorporation by TEC 1 (grey column), TEC 2 (black column) and HAECs (white column) cultured in EGM-2 in presence of 0.5% serum. (**B**) Proliferation of normal HAECs (white columns), TEC 1 (grey columns), and TEC 2 (black columns) in response to different concentrations of FCS or to FCS plus growth factors (GFs) added to medium for cell culture, evaluated after 72 h. Results are the mean ± SE of at least six experiments, *p* < 0.001 *vs*. HAECs. (**C**) Nitric oxide assay. Cells were incubated with vehicle alone, 1 μM acetylcholine, or 1 μM acetylcholine in the presence of 10^−3^ M of L-NAME. Activity of the endogenous NO system was controlled by stimulating cells with L-arginine, the substrate for NO synthase (data not shown). Experiments were repeated three times. NO values are expressed as cell absorbance at 540 nm, mean ± SE *p* < 0.001 *vs*. control. (**D**) PAF production from 1 × 10^6^ normal HAECs (white columns), TEC 1 (grey columns), and TEC 2 (black columns) incubated with vehicle or with 2 U/mL thrombin for 15 min at 37 °C. Data are the mean ± SE of three individual experiments; *p* < 0.001 *vs*. control (HAECs).

To determine the behaviour of TECs *in vitro*, we selected two representative cell lines, TEC 1 and TEC 2 (from chondrosarcoma and osteosarcoma biopsies respectively and positive to CD44, CD105, CD90 and CD146), for further functional and molecular studies. Both cell lines showed proliferation at low serum concentration (0.5%), and their growth rate was significantly higher than HAECs, even at the higher serum concentration and in the presence of serum plus growth factors known to represent optimal conditions for HAEC growth (Figure 4A,B). 

**Figure 5:**
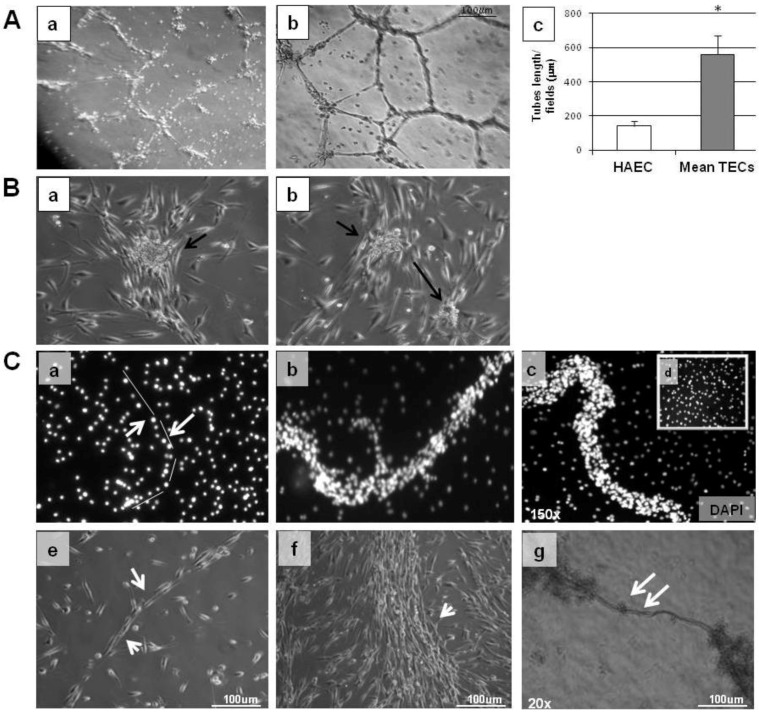
*In vitro* angiogenesis of TECs**.** Normal HAECs and TECs (5.0 × 10^4^ cells/well) were plated on growth factor-reduced Matrigel in EGM-2 containing plus 0.2% BSA and were observed after 3 h. (**A**), (**a**) Representative micrograph of the weak network of tubes formed by HAECs in the absence of serum. (**b**) Representative micrograph of the complete network of tubes formed by TEC 2 cell line persisting for several days (20×). (**c**) Bar graph of mean tube length x fields/µm of different TEC cell lines (TEC 1 and TEC 2) evaluated by computer analysis system in 3 different fields at 20× magnification in duplicate wells of two different experimental points (* *p* < 0.05). (**B**, **a**,**b**) Typical examples of TEC colonies. As indicated by arrows an endothelial colony consists of a central core of rounded cells surrounded by sprouting cells. (**C**) Direct observation of spontaneous tube-forming process by endothelial bone tumor cells cultured with stromal-derived growth factor (SDF-1). (**a**–**c**) TEC 1 spontaneous tube formation in presence of SDF-1 stained with DAPI at different time points as indicated at 150× magnification; (**a**) t = 0; (**b**) t = 30 min; (**c**) t= 1 h; (**d**) HAEC cells in presence of 10 ng/mL SDF-1 t = 1 h. (**e**–**g**) direct *in vitro* micrograph of the tube formation at different time points; (**e**) t = 0; (**f**) t = 30 min; (**g**) t = 1 h (20× magnification).

TEC 1 and TEC 2 were able to respond to acetylcholine and thrombin stimulation in terms of synthesis of NO and PAF, respectively (Figure 4C,D). To compare angiogenic properties of TECs and normal ECs we performed tube formation assays (Figure 5A). 

In contrast to HAECs, TECs showed the ability to make tubes when plated onto basal Matrigel in only a few minutes. TECs formed structures that persisted for several days in 0% serum (Figure 5A panel b) unlike normal HAECs, which rapidly undergo apoptosis when plated onto Matrigel in the absence of serum (panel a). At 4 h, under conditions providing optimal branch formation by HAECs (5% FCS, tube length 170 ± 10 μm × field), TECs showed enhanced formation of branches (570 ± 20 μm tube length × field; * *p* < 0.05). No differences in the spontaneous random motility of TECs were observed compared with normal HAECs (data not shown). In addition, TECs were able spontaneously to migrate one to another and organize themselves into colony formation units (Figure 5B), while in presence of stromal-derived growth factor (SDF-1) they were able to form spontaneous tubular structures (Figure 5C). Together, these data indicate that TECs not only present an endothelial phenotype but also behave as ECs.

## 4. Discussion

Here, we introduce for the first time the concept of bone tumor endothelial heterogeneity that arises from a complex heterotypic cross-talk interplay among osteoblasts, endothelium and environment [21,22]. In the present study, we isolated and characterized a population of endothelial bone tumor cells from 13 tumor biopsies with different grade and histology. TECs selected by CD31 antibody as already reported [23] lost this antigen early *in vitro* [24]. We selected from different histotypes an homogenous population expressing both endothelial and mesenchymal markers. Thus, TECs were positive, with different grade, to endothelial markers such as CD105, CD146, von Willebrand factor together with CD90, and, in addition, were strongly positive to CD44 antigen. Cells spontaneously showed an outgrowth forming colony units, normally associated with late endothelial progenitor cells (EPC) in other published studies [25,26]. Furthermore, recent observations have demonstrated that vascular endothelium itself contains highly proliferative endothelial colony-forming cell subpopulations (ECFCs), responsible for forming vessel lumens [27,28]. In contrast, no studies to date have determined whether these cells might represent the majority of angiogenesis response in tumors. Our functional studies demonstrated that bone CD31^+^ TECs are committed to forming vessels and, in contrast to normal ECs, and in presence of SDF-1, were able to differentiate into tubular structures in a few minutes. Although, these cells are a subpopulation of ECs present in the tumor, we observed that they are independent from tumor environment and VEGF to form vessels, consistent with previous reports [29,30], thus suggesting a permanent genetic change in their pro-angiogenic phenotype. The origin of TECs is still unknown; however, the presence of both endothelial and mesenchymal antigens indicates that they may originate from mesenchymal cells. EPCs and mesenchymal stem cells have the ability to trans-differentiate from one lineage to the other via an endothelial-to-mesenchymal process [31,32,33] however, it has been reported to have anticancer activity through the inhibition of angiogenesis [34,35]. The findings that TECs have stem cell features such as self renewal, migration capacity, longevity and normal karyotype but lack of main stem antigen such as CD133, suggest that TECs do not originate from tumor stem cells [7,36]. However, we cannot exclude that bone TECs derive from recruitment of EPCs following accumulation of genetic and epigenetic damage as reported by several studies [37]. We know that the present isolated TEC lines only partially represent bone TEC population because endothelium is heterogeneous in both tumor site and stage [36]. Further studies using different technical approaches and a better immunophenotyping will be needed to characterize other endothelial cell populations in order to understand tumor vessel complexity [37,38]. Here, we highlight for the first time that CD31^+^ bone TECs express a constitutive proangiogenic phenotype and have a different immunophenotype from normal endothelium, but have the same behaviour. We believe that TECs cultured from specific tumors are ideal tools to test genetic and chemical compounds in order to find angiogenic inhibitors on a high-throughput basis. In addition, cell-based assays using various TECs could be a rational approach to study ECs in tumor tissues. Finally, these findings may be of great importance in designing more tissue-specific antiangiogenic therapies for sarcoma and bone metastasis treatment or identifying novel markers to improve clinical outcomes [15,39].

## 5. Conclusions

Endothelial cells were in the past considered genetically stable and therefore good candidates for specific therapies because they were not expected to develop resistance. Here, we show that tumor endothelial cells have a particular proangiogenic phenotype. We believe that with better knowledge of the molecular and functional characteristics of tumor endothelium, can become a valid device to design appropriate antiangiogenic therapeutic approaches and to define novel tools to improve the predictive value of imaging techniques.
